# The role of fruit trees in reducing food insecurity and improving nutrition security of rural households: A case study of the KwaZulu-Natal province, South Africa

**DOI:** 10.1016/j.jafr.2025.101883

**Published:** 2025-06

**Authors:** Fortunate Nosisa Zaca, Unity Chipfupa, Temitope Oluwaseun Ojo, Lavhelesani Rodney Managa, Tafadzwanashe Mabhaudhi, Rob Slotow, Mjabuliseni Simon Cloapas Ngidi

**Affiliations:** a African Centre for Food Security, School of Agricultural, Earth and Environmental Sciences, College of Agriculture, Engineering and Science, University of KwaZulu-Natal, Private Bag X01, Scottsville, Pietermaritzburg, 3201, South Africa; b Centre for Transformative Agricultural and Food Systems, School of Agricultural, Earth and Environmental Sciences, College of Agriculture, Engineering and Science, University of KwaZulu-Natal, Private Bag X01, Scottsville, Pietermaritzburg, 3201, South Africa; c Human Sciences Research Council, Africa Institute of South Africa, 134 Pretorius Street, Pretoria, 0002, South Africa; d Department of Agriculture and Animal Health, School of Agriculture and Life Sciences, University of South Africa, 28 Pioneer Ave, Florida Park, Roodepoort, 1709, South Africa; e Department of Agricultural Economics, Obafemi Awolowo University, Ile-Ife, 220101, Nigeria; f Disaster Management Training and Education Centre for Africa, University of the Free State, Bloemfontein, 9301, South Africa; g Centre on Climate Change and Planetary Health, London School of Hygiene, Health and Tropical Medicine, Keppel Street, London, WC1E7HT, United Kingdom; h Centre for Functional Biodiversity, School of Life Sciences, College of Agriculture, Engineering and Science, University of KwaZulu-Natal, Private Bag X01, Scottsville, Pietermaritzburg, 3201, South Africa; i Department of Agricultural Extension and Rural Resource Management, School of Agricultural, Earth and Environmental Sciences, College of Agriculture, Engineering and Science, University of KwaZulu-Natal, Private Bag X01, Scottsville, Pietermaritzburg, 3201, South Africa

**Keywords:** Food insecurity, Nutrition security, Fruit trees, Rural households, Ordered logit model, Psychological capital

## Abstract

Despite calls to increase fruit consumption, food and nutrition security strategies often overlook the inclusion of fruit trees in the rural food systems. Hence, this study investigated the role of fruit trees in reducing food insecurity and improving nutrition security among rural households in the KwaZulu-Natal province, South Africa. Descriptive statistics, household food insecurity access scale (HFIAS), household food insecurity access prevalence (HFIAP), food consumption score (FSC), principal component analysis (PCA), and ordered logit model were used to analyze survey data from 305 households. The results showed that only 29.8% of the households were food secure, while the rest were either mildly (36.4%), moderately (27.9%), or severely (5.9%) food insecure. Moreover, 4.6% of the households consumed poor diets, 23.0% of the sampled households were at the borderline, and 72.5% consumed an acceptable diversity of food groups. The ordered logit model findings showed that growing fruit trees, consumption of wild fruits, household size, off-farm income, access to irrigation, access to training, livestock ownership, and psychological capital significantly influenced household food insecurity and nutrition security. The study recommends the implementation of awareness campaigns promoting the planting of fruit trees and the consumption of locally available wild fruits. There is a need for nutrition-related training programs and workshops to enhance awareness of the importance of growing and consuming fruits among rural households. The collective participation of the private sector, government, researchers, civil society organizations, policymakers, politicians, and farming rural households in building awareness is also recommended.

## Highlights

• Growing fruit trees reduces household food and nutrition insecurity.
• Promote planting fruit trees and consuming locally available wild fruits.
• Promote nutrition-related training programs and workshops.
• Establishment, rehabilitation, and revitalization of irrigation schemes.

## Graphical abstract

**Figure:**
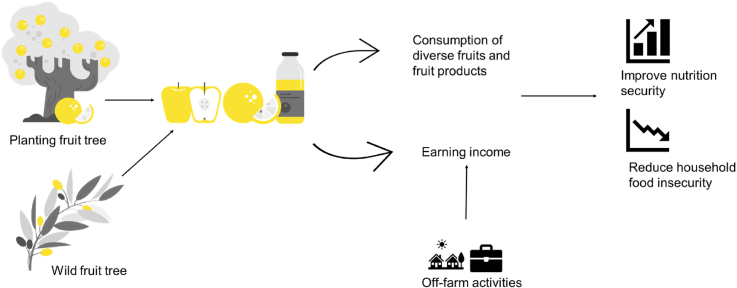

## 1 Introduction

The world has been struggling to achieve the United Nations' Sustainable Development Goals (SDGs) targets of eradicating hunger, ensuring constant access to safe, nutritious, and sufficient food for all people, and eliminating all forms of malnutrition [1]. High levels of food and nutrition insecurity are prevalent in sub-Saharan Africa, with over one-third of the population experiencing undernourishment [2]. About two billion people worldwide lack access to adequate food [3]. According to FAO et al. [4], more than 864 million people globally experienced severe food insecurity in 2023. That is, they spent an entire day or more without eating. Challenges such as the COVID-19 pandemic, economic instability, and international conflicts (e.g., the Russia-Ukraine war) have put the world off track to eradicating food insecurity and malnutrition in all its forms by 2030 [3,5]. Such challenges affect the capacity of food systems and cause supply chains to not function properly in African countries, including South Africa [6]. The Southern African Development Community (SADC) has been working to significantly decrease food and nutrition insecurity in selected African countries by 2025, and progress is still ongoing [7]. Hence, food and nutrition insecurity reduction is one of the top agenda items for the South African government. For example, the country's National Policy on Food and Nutrition Security of 2013 aims to utilize a multi-sectored approach to combat food and nutrition insecurity [8].

In South Africa, 12.9% of people experienced hunger in 2022 [9,10]. Although the country is food secure at a national level, it remains food insecure at the household level. For instance, 19.6% of South African households considered their access to food as inadequate or severely inadequate in 2022 [9,10]. Moreover, about 10 million people in South Africa experienced severe food insecurity between 2021 and 2023 [4]. Rural households usually experience higher rates of food and nutrition insecurity and different forms of malnutrition than urban households [3]. According to McMullin et al. [11], malnutrition is mainly caused by a low-quality diet with inadequate consumption of fruits and vegetables. Thus, it is important to ensure that all individuals have access to adequate and nutritious food produced in an environmentally and socio-culturally sustainable way [12,13]. In the present context of climate change, continuous loss of species and genetic diversity, soil degradation, rising urbanization, social conflict, and extreme poverty, collective and effective action is required to address food and nutrition insecurity [5,14].

Tree restoration and improved forest management are key strategies to reduce food and nutrition insecurity [15]. Hence, tree resources need to be incorporated into policies because they contribute to attaining the six pillars of food security (availability, access, utilization, stability, agency, and sustainability) [11,13,16,17]. Trees contribute significantly to food and nutrition insecurity reduction through the direct provision of food and indirectly through energy for cooking. Edible tree resources have been reported to decrease food insecurity through improved dietary diversity [18] and providing nutritional diets [19], particularly for rural households [20]. For example, nuts and fruits harvested from trees are crucial sources of micronutrients in many rural communities because they are easily accessible, inexpensive, and nutritious [11,21,22]. Tree products also contribute indirectly through income provision [12,16].

Though several studies explored the role of tree resources in addressing food and nutrition insecurity [e.g., 11,16], there is insufficient empirical research on the role of fruit trees on food insecurity and nutrition security. Some of the limited studies known to the authors that have attempted to examine the contribution of trees to food and nutrition insecurity are [13,16], and [17]. The first two studies are based on a comprehensive review of the existing literature. The authors did not use primary data on the actual contribution of trees. Using detailed data (collected utilizing a pre-tested and structured questionnaire) is essential to understand the actual role of fruit trees. Moreover, although [17] used an empirical model in their study, the authors did not include the contribution of fruit trees to food groups consumed by each household in their analysis. Therefore, this study aimed to investigate the role of fruit trees in food insecurity and nutrition security to understand the full scope of food access and dietary quality among rural households. Growing fruit trees and wild fruit consumption were used as proxies to represent the role of fruit trees. Hence, the specific objectives of this study were to: (1) assess the association between growing fruit trees and wild fruit consumption (independent variables) and household food insecurity status (dependent variable); and (2) examine the relationship between growing fruit trees and wild fruit consumption (independent variables) and household nutrition security status (dependent variable). The research question was: what is the association between the two independent variables representing the role of fruit trees (growing fruit trees and wild fruit consumption) and the household food insecurity and nutrition security status? The study hypothesized that growing fruit trees and wild fruit consumption are negatively associated with household food insecurity status and positively associated with household nutrition security status.

Another novel aspect of this study is that it adopted a definition of food security with six pillars. Including agency and sustainability dimensions expands a four-pillar framework for food security to a six-dimensional one to address rising inequities within food systems. A six-pillar framework was proposed by the High Level Panel of Experts on Food Security and Nutrition (HLPE-FSN) and accepted by the Food and Agriculture Organization and other United Nations agencies in the State of Food Security and Nutrition in the World 2021 report [23]. However, this framework is not directly used for measuring food security in this study. Instead, it informs the conceptual framework by highlighting external factors and broader livelihood outcomes associated with fruit tree planting and utilization, with particular emphasis on the agency and sustainability pillars. Agency refers to the ability of individuals and communities to exercise their voices and participate in their local food systems [23,24]. Agency can be improved through equitable access to practical training, agricultural inputs, extension services, and arable land and through collective participation in shaping food systems and institutional frameworks (e.g., involvement of farmers), especially in vulnerable communities. Sustainability refers to resilient food systems that maintain natural, social, and economic systems and fulfil the food needs of current and future generations [23]. Policy initiatives such as the SDGs also emphasize the significance of adopting sustainable food systems such as climate-smart agriculture [25,26].

## 2 Conceptual framework

**Fig. 1:**
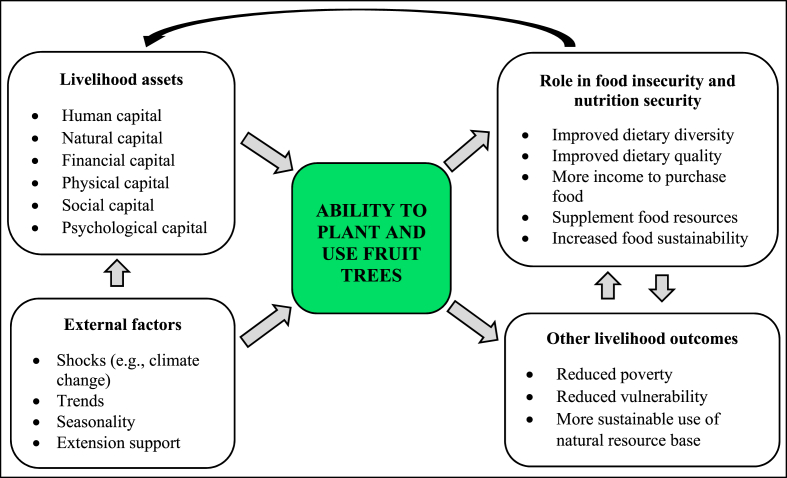
The conceptual link: role of fruit trees on food insecurity and nutrition security.

**Source:** Authors' compilation

Fig. 1 presents the conceptual framework with its dimensions and role pathways. The conceptual framework in this study argues that the role of livelihood assets (human, financial, physical, natural, social, and psychological capitals) and external factors such as climate change and extension support must be accounted for in explaining the contribution of fruit trees in addressing food insecurity and improving nutrition security. It is grounded in resilience theory, which suggests that an individual's ability to withstand adversity and adapt to challenges plays a key role in their agricultural decision-making [27]. The framework assumes that rural households with adequate access to key livelihood assets are better equipped to pursue various agricultural activities, such as planting fruit trees, which contribute to food and nutrition security. The psychological capital, which defines an individual's mindset, perceptions, and behaviour [28], is a critical asset in this context. The psychological capital comprises four constructs, i.e., self-confidence, hope, optimism, and resilience [28]. It is assumed that despite the difficulties, confident rural farming households believe in their capacity to grow and nurture fruit trees. Hope affords such households the willpower to persevere and proactively devise alternative solutions when facing challenges. Optimism enables them to have positive expectations about the future of agroforestry. As a venture, setbacks or failures in planting fruit trees are common due to several adverse conditions. The households' resilience in the face of such adversities will thus be critical. If a household does not possess or have access to these assets, the result is failure to plant and use fruit trees to address food and nutrition security challenges [29].

The framework further assumes that external factors such as climate change and extension support play a significant role in influencing a household's ability to plant and use fruit trees [30]. Extension support strengthens the agency pillar of food security through equipping farming rural households with the knowledge, skills, and resources required to make informed decisions about food production and consumption. Specifically, extension services can play a crucial role in promoting tree planting by providing technical training on species selection, planting techniques, and sustainable management practices. This, in turn, can empower rural households to integrate fruit trees into their farming systems, potentially contributing to improved food and nutrition security. The external factors can also impact an individual's ability to possess livelihood assets, thus, can either promote or hinder the adoption of fruit tree planting. For instance, climate change can hinder the success of fruit tree cultivation.

In alignment with this study's specific objectives, the framework hypothesizes two plausible pathways through which fruit trees contribute to reducing food insecurity and improving nutrition security. When the fruits from these trees are consumed, they can enhance dietary diversity and improve nutrition security (direct contribution). Income generated from the sales of fruits can be utilized to buy food items to supplement the household's diet and contribute to improved food access, thereby reducing food insecurity (indirect contribution) [11,16,31]. Fruit trees can contribute to the attainment of food security pillars in the following ways: by providing a supplement to staple food all year long (availability), in periods of food shortage (stability), through increased dietary quality (utilization); and as a source of direct dietary improvement or income to purchase food (access) [16]. Other livelihood outcomes that arise from planting and using fruit trees include reduced poverty, reduced vulnerability, and improved sustainable use of natural resources. Households with diversified food sources are better equipped to withstand economic and environmental shocks, enhancing their overall resilience [18]. Moreover, fruit trees provide long-term environmental benefits, such as improved soil conservation and carbon sequestration [32,33]. They also contribute to the food sustainability pillar because some species are more drought-resistant and pest-tolerant than annual crops, thus, providing food in dry periods when other food sources are unavailable [11].

## 3 Research methodology

### 3.1 Study area, sampling strategy, and data collection

**Fig. 2:**
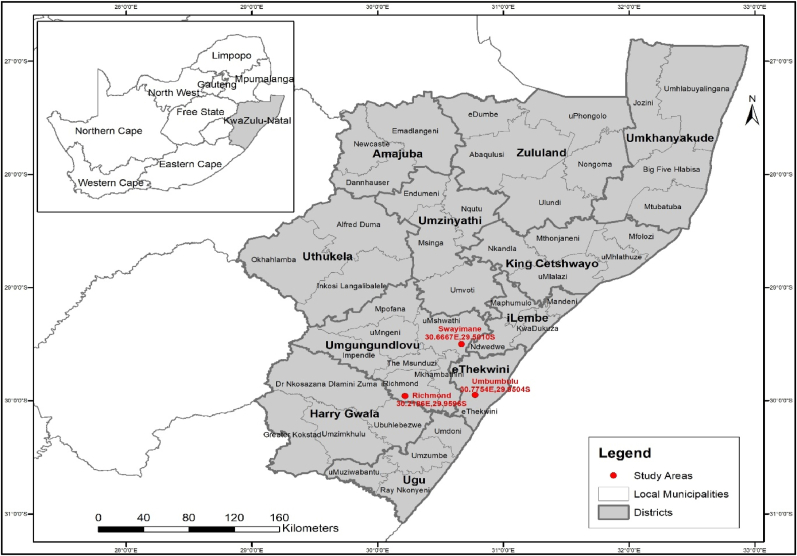
Map showing the KwaZulu-Natal province of South Africa.

The study focused on rural households in the KwaZulu-Natal province, South Africa (Fig. 2). The province has the second-highest population of approximately 12.4 million [10]. The province's average temperature increases above 25 °C in summer and falls below 20 °C during winter [34]. Moreover, the average annual rainfall is about 800 mm [35]. The survey was conducted in three study sites: Swayimane (30.6667°E, 29.5010°S), Umbumbulu (30.7754°E, 29.9504°S), and Richmond (30.2186°E, 29.9596°S). Swayimane and Richmond are in the uMgungundlovu District Municipality, while Umbumbulu is in the eThekwini Metropolitan Municipality. Most households in the study sites are involved in various farming activities such as crop, fruit, and livestock production. The majority of household members rely on social grants as their source of income [9].

A multistage sampling approach was employed to carry out the survey. The first stage involved the purposive selection of the KwaZulu-Natal province due to its high levels of unemployment, poverty, and food insecurity, particularly among rural households [9]. This was done purposively to align the study with the South African government's plan to reduce poverty and improve food security. The second stage was the identification of municipalities with households engaged in small-scale farming activities. The municipalities were also selected based on the presence of homestead and wild fruit trees. In the third stage, rural households were randomly selected from the three study sites for interviews. The initial target sample size for this study was set at 300 rural households, based on practical factors such as time, available resources, and accessibility of households during data collection. However, the final number of interviews conducted was 317, which was influenced by logistical feasibility and the availability of households during the fieldwork process. After data cleaning, only 305 questionnaires were valid and utilized for the analysis: Swayimane (92), Umbumbulu (103), and Richmond (110). This sample size is reasonably large and considered sufficient for conducting statistical analysis [36].

The data were collected from September 2022 to October 2022 by trained enumerators who spoke the local language (IsiZulu). Research ethical clearance was obtained from the Humanities and Social Sciences Research Ethics Committee (HSSREC) of the University of KwaZulu-Natal (protocol reference number: HSSREC/00003793/2022). Moreover, all the ethical requirements such as informed consent and confidentiality, were observed throughout the study. A structured and pre-tested questionnaire was used to collect data. The questionnaire encompassed questions about socio-economic and demographic characteristics, livelihood assets, fruit trees, food insecurity, food consumption, and agricultural production. Following previous studies [29,37], the questionnaire also encompassed five-point Likert scale statements to measure the level of psychological capital endowment. However, contrary to these studies, this study used a set of scenario-based questions to measure psychological capital and generate data that are close to the revealed preference approach. The data collection took place during the fruiting season of the following fruit trees: banana, peach, lemon, kei apple, mulberry, mango, and papaya. Households grew different types of fruit trees, ensuring fruits were produced throughout different seasons. This diversity allowed for consistent fruit availability across households, which justified their inclusion in the analysis and aligned with the food security indicators assessed during the 30-day recall period. In addition, focus group discussions were conducted to complement information collected during the household survey.

### 3.2 Data analysis

Descriptive statistics such as percentages, means, standard deviations (Std. Dev.), and standard errors (Std. Err.) were used to summarize the data. A chi-square (Chi^2^) test was conducted to determine whether there were statistically significant differences between the three study sites. Food insecurity status was measured using the household food insecurity access scale (HFIAS) and household food insecurity access prevalence (HFIAP). The food consumption score (FCS) was used to assess dietary intake or nutritional status. The principal component analysis (PCA) was employed to create psychological capital indices. The role of fruit trees in food insecurity and nutrition security was evaluated using the ordered logit model. The International Business Machines (IBM) Statistical Package for Social Sciences (SPSS) version 28 and STATA SE version 17 were used for statistical data analysis. Moreover, bar charts were created using Microsoft Excel 2019 to organize and summarize the data.

#### 3.2.1 Food insecurity measurement

The HFIAS was used to evaluate the status of food insecurity among rural households. It has been widely used to determine the household food insecurity status [e.g., 8,38,39,40,41]. The HFIAS is a self-reported food insecurity measure based on a methodology developed by the Food and Nutrition Technical Assistance (FANTA) Project, which was funded by the United States Agency for International Development (USAID). Its aim is to categorize households into different food (in)security levels [8,42]. The validity and reliability of HFIAS in measuring household food insecurity were confirmed by Knueppel et al. [43] and Becquey et al. [44]. The HFIAS comprises nine frequency-of-occurrence questions capturing the three dimensions of household food insecurity: anxiety and uncertainty about food access, insufficient food quality (includes dietary diversity, nutritional adequacy, and preferences), and insufficient food intake and the physical consequences or hunger [45]. The HFIAS score determines the household food insecurity level. According to Coates et al. [45], the score is a continuous measure of the degree of food insecurity (access) in the past four weeks (i.e., 30 days). The HFIAS score variable was calculated for each household by adding the codes for each frequency-of-occurrence question about food access at the household level. Each of the nine questions has a maximum score of three. Therefore, the minimum score for a household is zero (more food secure) and the maximum score is 27 (more food insecure) [45]. The average HFIAS score is calculated using the following equation:

(1) $$Average\;HFIAS\;score=\frac{Sum\;of\;HFIAS\;scores\;in\;the\;sample}{Total\;number\;of\;HFIAS\;scores\;in\;the\;sample\;\left(i.e.,households\right)}$$

Moreover, the HFIAP indicator was used to illustrate the prevalence of household food insecurity. It categorizes households into four levels of food (in)security status, namely, food secure, mildly food insecure, moderately food insecure, and severely food insecure [45]. These four categories were used as a dependent variable in this study. For more details on the computation of HFIAP and the definition and calculation of each household food insecurity (access) category see Coates et al. [46:19–21].

#### 3.2.2 Nutritional adequacy measurement

According to D'Haese et al. [42], the HFIAS and HFIAP measurements do not give a complete picture of the food security and nutrition status of households and individuals. Therefore, this study also used the FCS as an indicator of dietary intake. Previous studies have shown that the FCS is significantly associated with nutritional status indicators and can be used as a proxy for nutritional adequacy [[46], [47], [48], [49], [50]]. The FCS is based on dietary diversity, food frequency, and the relative nutritional importance of various food groups consumed by a household over a seven-day recall period [51]. Thus, it is preferred to other indicators solely focusing on food diversity [46]. The nine food groups are main staples, pulses, vegetables, fruits, meat and fish, milk, sugar, oil, and condiments. Each food group is multiplied by its weight. The FCS is then calculated by summing the nine weighted food groups consumed by each household in the past seven days before the survey [51]. While a higher score indicates nutrition security, a lower score indicates nutrition insecurity. For more details on the food items included in each food group see WFP [52:8]. Following Hasanah et al. [46] and Isaura et al. [47], this study used the FCS to categorize households into three food consumption groups depending on the score value: poor (0–21), borderline (21.5–35), and acceptable (greater than 35). These three categories were used as a dependent variable in this study. The number of meals consumed by a household per day was also used as an indicator of food consumption status [42].

#### 3.2.3 Ordered logit model

The ordered logit model was used to examine the role of fruit trees on household food insecurity and nutrition security. The dependent variable of this model is categorical and ordered [52]. The model is used when the regressand has more than two ordered categories, and the value of each category is higher than the previous one [[53], [54], [55]]. The dependent variable used to measure food insecurity in this study has four ordered categories (i.e., 1 = food secure; 2 = mildly food insecure; 3 = moderately food insecure; and 4 = severely food insecure). Moreover, the dependent variable measuring nutrition security has three ordered categories (i.e., 1 = poor; 2 = borderline; and 3 = acceptable). Therefore, following previous studies with a similar dependent variable [53,54,56,57], the ordered logit model was selected for regression analysis. Although both ordered logit and probit models are the most appropriate for analyzing ordinal survey data, it is argued that selecting between the two models is a matter of choice because they both usually give the same results [[58], [59], [60]].

The difference between the two models is that the ordered logit assumes a logistic distribution of the error term, while the ordered probit assumes a normally distributed error term [61]. Moreover, the multinomial logit or probit models were inappropriate for the analysis in this study because they fail to account for the ordinal nature of the dependent variable [62]. In the ordered logit model there is an observed ordinal variable (Y) which is a function of an unobserved latent variable $\left(Y^{*}\right)$. The latent variable has numerous threshold points and its properties are useful and intuitive [54,63]. In this study, the ordered logit model was constructed on an unobservable latent random variable as follows [53,54,62]:

(2) $$Y_{i}^{*}=X_{i}^{\prime }\beta +\varepsilon_{i}\;\left(i=1,2,3,\ldots ,n\right)$$

where $Y_{i}^{*}$ is the unobservable latent random variable with more than two ordered categories and denotes the level of food insecurity in household i, $X_{i}^{\prime }$ is a vector of explanatory variables affecting food insecurity in household i, β is a vector of parameters to be estimated, and $\varepsilon_{i}$ is a random error term which is assumed to be logistically distributed. The relationship between the observed ordinal variable $\left(Y_{i}\right)$ and unobserved latent variable $\left(Y_{i}^{*}\right)$ is described from the food insecurity model as follows:

(3) $$\begin{array}{c} Y_{i}=1\;if\;0<Y_{i}^{*}\leq \mu_{1}\;\left(Food\;secure\right)\\ Y_{i}=2\;if\;\mu_{1}<Y_{i}^{*}\leq \mu_{2}\;\left(Mildly\;food\;insecure\right)\\ \begin{array}{c} Y_{i}=3\;if\;\mu_{2}<Y_{i}^{*}\leq \mu_{3}\;\left(Moderately\;food\;insecure\right)\\ \;Y_{i}=4\;if\;Y_{i}^{*}>\mu_{3}\;\left(Severely\;food\;insecure\right) \end{array} \end{array}$$

where $\mu_{1}$ to $\mu_{3}$ are unknown parameters or threshold points to be estimated with β. The relationship between the observed ordinal variable $\left(Y_{i}\right)$ and unobserved latent variable $\left(Y_{i}^{*}\right)$ is described from the nutrition security model as follows:

(4) $$\begin{array}{c} Y_{i}=1\;if\;0<Y_{i}^{*}\leq \mu_{1}\;\left(Poor\right)\\ Y_{i}=2\;if\;\mu_{1}<Y_{i}^{*}\leq \mu_{2}\;\left(Borderline\right)\\ \;Y_{i}=3\;if\;Y_{i}^{*}>\mu_{2}\;\left(Acceptable\right) \end{array}$$

where $\mu_{1}$ to $\mu_{2}$ are unknown parameters or threshold points to be estimated with β. The probability of observing a particular response (j) for household i is expressed as follows [56]:

(5) $$\begin{array}{c} Ρrob\left(Y_{i}=j\right)=Ρrob\left(\mu_{j-1}<Y_{i}^{*}\leq \mu_{j}\right)=Ρrob\left(\mu_{j-1}-X_{i}^{\prime }\beta <\varepsilon_{i}\leq \mu_{j}-X_{i}^{\prime }\beta \right)\\ \begin{array}{c} =F\left(\mu_{j}-X_{i}^{\prime }\beta \right)-F\left(\mu_{j-1}-X_{i}^{\prime }\beta \right)\\ =\frac{e^{\left(\alpha_{j}+X_{i}^{\prime }\beta \right)}}{1+e^{\left(\alpha_{j}+X_{i}^{\prime }\beta \right)}} \end{array} \end{array}$$

where j denotes the ordered categories of the dependent variable (e.g., 1, 2, 3, and 4), F denotes the standard logistic cumulative distribution function, and $\alpha_{j}$ is the intercept for j logit. The Hosmer–Lemeshow, Lipsitz, and likelihood ratio tests were used to evaluate the goodness of fit of the ordered logit model in this study. The statistically insignificant Hosmer–Lemeshow and Lipsitz test values (p-value > 0.10) indicate a good fit of the model [64]. On the contrary, a statistically significant likelihood ratio Chi^2^ test value (p-value < 0.05) indicates a good fitting ordered logit model [65]. Moreover, the Durbin-Wu-Hausman test was performed to detect the presence of endogeneity between the following variables: education, off-farm income, gender, training, irrigation, livestock ownership, and hope. A statistically insignificant Chi^2^ test value (p-value > 0.10) indicates the absence of endogeneity. The Approximate Likelihood-Ratio test of proportionality of odds was performed to assess whether the proportional odds assumption holds. A statistically insignificant Chi^2^ test value (p-value > 0.10) indicates that the proportional odds assumption is not violated. The variance inflation factor (VIF) was also calculated to test for multicollinearity among explanatory variables. The average VIF below the threshold value of 10 implies the absence of multicollinearity [36]. Table 1 shows the description of the explanatory variables used in the ordered logit model. The PCA-derived psychological capital indices (i.e., self-confidence, hope, optimism, and resilience) were included as the explanatory variables. The results for these indices are presented in Table A1. For more details on the PCA technique, see [66].

**Table 1:** Description of explanatory variables, their means, standard deviations, and percentages.

Variable	Description	Mean	Std. Dev.	%
**Continuous variables**				
Age	Household head age (Years)	61.83	14.05	–
Household size	Household size (Number)	5.88	2.85	–
Education	Household head education level (Years of schooling)	5.48	4.90	–
Off-farm income	Log of the annual income from non-farm activities	10.88	0.86	–
**Dummy variables**				
Growing fruit trees	Household involved in growing fruit trees (1 = Yes; 0 = Otherwise)	–	–	89.8
Wild fruit consumption	Consumption of wild fruits per household (1 = Yes; 0 = Otherwise)	–	–	34.8
Gender	Gender of household head (1 = Male; 0 = Otherwise)	–	–	42.0
Training	Access to agricultural training (1 = Yes; 0 = Otherwise)	–	–	35.1
Irrigation	Access to water for irrigation purposes (1 = Yes; 0 = Otherwise)	–	–	46.9
Livestock ownership	Ownership of livestock per household (1 = Yes; 0 = Otherwise)	–	–	80.3

Self-confidence		–	–	–
Hope	Psychological capital indices computed using PCA	–	–	–
Optimism		–	–	–
Resilience		–	–	–

## 4 Results and discussion

### 4.1 Household fruit production

The results showed that most rural households planted fruit trees (Table 2). The Chi^2^ test results indicated that the production of lemons was statistically different across the selected study locations at the 10% significance level. The most common fruit type at Richmond was peach (72.7%). A few rural households reported that they produced kei apple (3.6%), papaya (3.3%), and naartjie (3.0%). Some households (3.6%) generated income from the sale of fruits. According to Bhebhe et al. [17], encouraging rural households to plant more trees on their homesteads and sell tree products can sustain their household income and improve food security. The findings showed that 9.2% of the sampled households used fruit trees for medicinal purposes. During the survey, some respondents indicated that they use peach and guava leaves to treat stomach aches and diarrhoea, respectively. This is in line with Omotayo and Aremu [13], who reported that fruit trees play a crucial role in the healthcare system of rural communities that still rely on traditional medicine. Ojha et al. [67] also emphasized the importance of awareness of agriculture systems that improve food-medicine security and avoid malnutrition among rural households.

**Table 2:** Fruits produced by sampled households (%).

Variable	Swayimane	Umbumbulu	Richmond	Total	Chi^2^ - test
Households producing fruits	88.0	94.2	87.3	89.8	3.24

Type of fruit produced					
*Banana*	29.3	65.0	24.5	39.7	42.33∗∗∗
*Guava*	42.4	12.6	38.2	30.8	24.57∗∗∗
*Peach*	55.4	44.7	72.7	58.0	17.57∗∗∗
*Orange*	22.8	21.4	29.1	24.6	1.94
*Lemon*	35.9	29.1	20.9	28.2	5.61∗
*Kei apple*	7.6	1.9	1.8	3.6	6.07∗∗
*Mulberry*	9.8	8.7	23.6	14.4	11.87∗∗∗
*Apple*	12.0	9.7	8.2	9.8	0.81
*Mango*	5.4	37.9	10.9	18.4	40.47∗∗∗
*Avocado*	32.6	38.8	6.4	25.2	33.50∗∗∗
*Naartjie*	6.5	1.0	1.8	3.0	6.00∗∗
*Papaya*	1.1	5.8	2.7	3.3	3.61

Main reason for producing fruits					
*Earn income*	5.4	4.9	0.9	3.6	3.65
*Consumption*	87.0	94.2	87.3	89.5	3.61
*Medicinal*	13.0	7.8	7.3	9.2	2.37

**Note**: ∗∗∗, ∗∗, and ∗ indicate the level of significance at 1%, 5%, and 10%, respectively; Multiple responses were allowed.

### 4.2 Household food insecurity access scale (HFIAS) results

The results showed that 59.3% of the interviewed households experienced anxiety and uncertainty about food access (Table 3). This is in line with Bhebhe et al. [17], who reported that more than 50.0% of households in KwaZulu-Natal were worried they would not have enough food in the past four weeks. The percentage of households who ate a limited variety of foods and some non-preferred foods often was 11.5% and 11.1%, respectively. Moreover, 15.4% of households rarely ate a smaller meal than required. Fewer households (3.9%) indicated that they sometimes went to sleep hungry due to inadequate quantities of food.

**Table 3:** Food insecurity conditions of sampled households in the past 30 days (%).

Variable	Yes	Frequency-of-occurrence		
		Rarely	Sometimes	Often
Worried that the household would not have enough food	59.3	24.6	25.9	8.9
Unable to eat preferred kinds of foods because of a lack of resources	63.0	21.6	29.8	11.5
Ate a limited variety of foods due to a lack of resources	59.3	23.9	23.9	11.5
Ate some non-preferred foods due to a lack of resources to obtain other food types	58.4	19.0	28.2	11.1
Ate a smaller meal than needed because there was not enough food	42.0	15.4	18.7	7.9
Ate fewer meals in a day because there was not enough food	36.4	14.1	16.4	5.9
No food to eat of any kind because of a lack of resources to get food	19.7	8.2	7.5	3.9
Went to sleep at night hungry because there was not enough food	10.2	4.3	3.9	2.0
Spent the whole day and night without eating anything because there was not enough food	7.2	3.3	3.0	1.0

Table 4 shows that the households in Richmond had a higher average HFIAS score (7.01), followed by those in Swayimane (6.95) and then Umbumbulu (5.26). The average score for the total sample was 6.40, indicating that most households in the study area were mildly food insecure [45]. This is lower than the average HFIAS score of 8.8 reported by [9] for the KwaZulu-Natal province, suggesting that food insecurity in Swayimane, Umbumbulu, and Richmond is somewhat less severe compared to the provincial average. This may imply that certain regional factors, such as local farming practices, community support systems, and socio-economic conditions, are contributing to the reduction of food insecurity in these study areas. Therefore, future studies need to identify the exact underlying causes of the different HFIAS scores across regions.

**Table 4:** The average household food insecurity access scale (HFIAS) score.

Variable	Swayimane		Umbumbulu		Richmond		Total	
	Mean	Std. Dev.	Mean	Std. Dev.	Mean	Std. Dev.	Mean	Std. Dev.
Average HFIAS score	6.95	6.44	5.26	5.55	7.01	6.76	6.40	6.31

### 4.3 Household food insecurity access prevalence (HFIAP) results

**Fig. 3:**
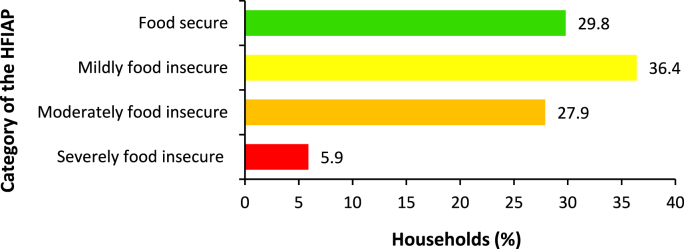
Household food insecurity access prevalence categories.

**Source:** Authors' own work

The HFIAP analysis showed that 70.2% of the households were food insecure (Fig. 3). This is in line with [9], who found that 70.4% of the households in the KwaZulu-Natal province experienced food insecurity. The food secure households (29.8%) rarely worried about not having enough food and experienced none of the other food insecurity conditions in the past four weeks. The mildly food insecure households (36.4%) worried about not having enough food sometimes or often, or were unable to eat preferred foods, or rarely consumed a monotonous diet and undesirable foods. The moderately food insecure households (27.9%) ate a monotonous diet and some non-preferred foods sometimes or often and/or reduced the size and number of meals rarely or sometimes. That is, they sacrificed the quality and quantity of foods consumed. Moreover, the severely food insecure households (5.9%) had to reduce the meal size or number of meals consumed often, and/or experienced the three most severe conditions (i.e., ran out of food, went to bed hungry, or spent a whole day and night without eating anything). The overall results indicate that most of the sampled households were food insecure.

### 4.4 Food consumption score (FCS) results

Table 5 shows that the food groups consumed by most households in the past seven days before the survey were condiments (100.0%), main staples (99.7%), oil (99.0%), vegetables (98.7%), and meat and fish (93.4%). The consumption of milk and other dairy products was relatively low (16.1%). Most households indicated that they consumed milk in small amounts (i.e., added to tea or coffee). Following WFP [51], small amounts of milk were treated as condiments in this study. The results also showed that households obtained their food items in various ways. For example, 42.0% of the households indicated that some vegetables consumed in the week were produced from their own farmland, while 89.8% reported purchasing some. These results illustrate that agricultural production is a source of livelihood for some rural households. A few households sourced meat and fish from the forest through hunting (0.7%) and river through fishing (2.6%). This indicates that forests and fisheries provide a limited contribution to the food groups consumed by rural households. Only 0.3% of the sampled households reported obtaining milk and/or maas (fermented milk) from their own cattle. This is in line with Xulu and Naidoo [68], who indicated that few rural households in KwaZulu-Natal are involved in small-scale dairy farming. Moreover, 11.8% of households obtained fruits from other sources such as events (e.g., funerals and weddings) and donations or gifts.

**Table 5:** Food groups consumed by sampled households in the past seven days and their sources (%).

Food group	Yes	Source food obtained from				
		Own production	Purchased	Forest	River	Other sources
Main staples	99.7	17.7	98.7	–	–	1.6
Pulses	63.6	8.5	54.4	–	–	1.3
Vegetables	98.7	42.0	89.8	–	–	3.0
Fruit	73.8	14.4	63.9	–	–	11.8
Meat and fish	93.4	14.4	90.2	0.7	2.6	6.6
Milk	16.1	0.3	15.7	–	–	–
Sugar	89.8	–	87.9	–	–	3.3
Oil	99.0	–	99.0	–	–	0.3
Condiments	100.0	–	100.0	–	–	0.3

**Note:** Multiple responses were allowed.

The average FCS in Swayimane, Umbumbulu, and Richmond was 45.16, 44.27, and 43.36, respectively (Table 6). The overall average FCS was 43.36, implying that most households consumed acceptable diets. The results also showed that the sampled households commonly consumed 2.98 meals per day on average. According to Ibe et al. [69], the standard number of meals per day recommended by nutrition experts is three (i.e., breakfast, lunch, and dinner).

**Table 6:** The average household food consumption score and number of meals consumed per day.

Variable	Swayimane		Umbumbulu		Richmond		Total	
	Mean	Std. Dev.	Mean	Std. Dev.	Mean	Std. Dev.	Mean	Std. Dev.
Average FCS	45.16	16.94	44.27	11.36	40.99	12.02	43.36	13.58
Average number of meals eaten per day	2.96	0.63	3.07	0.60	2.91	0.46	2.98	0.56

**Fig. 4:**
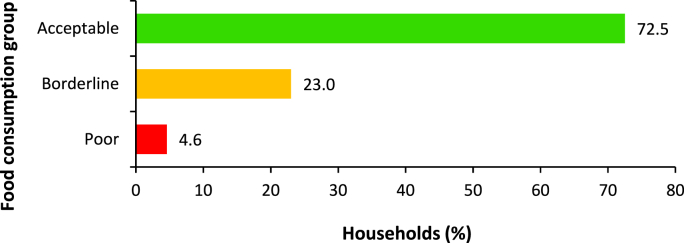
Categories of household food consumption groups.

**Source:** Authors' own work

Most rural households (72.5%) consumed an acceptable diversity of food groups in the past seven days (Fig. 4). Simelane et al. [9] also found the same. Their study showed that about 75.9% of households in the KwaZulu-Natal province consumed an acceptable number of food groups. The results also showed that while 23.0% of households were at the borderline, 4.6% consumed poor diets. This shows a need for educational programs on food consumption among rural households to improve the consumption of acceptable diets. It is also important to investigate the underlying reasons for inadequate consumption of food groups, as constraints related to availability or access would require interventions focused on addressing these structural barriers, alongside educational initiatives, to ensure a comprehensive solution.

### 4.5 Ordered logit model results

The ordered logit model was used to investigate the role of fruit trees on food insecurity and nutrition security of rural households (Table 7). The likelihood ratio Chi^2^ test value supported the existence of a relationship between the dependent variable and independent variables in both regression models (p-value = 0.000). The statistically insignificant Hosmer-Lemeshow (p-value = 0.732) and Lipsitz (p-value = 0.200) test values indicated a good fit of the model. Thus, this study accepted the null hypothesis that the ordered logit model fits the data well. The Durbin-Wu-Hausman test results (p-value = 0.988) indicated no evidence of endogeneity. Hence, this study accepted the null hypothesis that the variables are exogenous. The statistically insignificant Approximate Likelihood-Ratio test value (p-value = 0.138) indicated that the proportional odds assumption was not violated, confirming the appropriateness of the ordered logit model. Moreover, the average VIF was 1.20, indicating the absence of multicollinearity among the explanatory variables.

**Table 7:** The role of fruit trees on household food insecurity and nutrition security: Ordered logit model results.

Food insecurity model							Nutrition security model				
Variables	Coef.	Std. Err.	Marginal effects				Coef.	Std. Err.	Marginal effects		
			FS	Mildly FI	Moderately FI	Severely FI			Acceptable	Borderline	Poor
Growing fruit trees	−1.077∗∗∗	0.386	0.190∗∗∗	0.005	−0.140∗∗∗	−0.055∗∗	1.008∗∗	0.431	0.171∗∗	−0.129∗∗	−0.042∗∗
Wild fruit consumption	0.892∗∗∗	0.258	−0.158∗∗∗	−0.004	0.116∗∗∗	0.046∗∗∗	0.031	0.313	0.005	−0.004	−0.001
Age	−0.004	0.010	0.001	0.000	0.000	0.000	−0.007	0.012	−0.001	0.001	0.000
Gender	−0.303	0.232	0.054	0.001	−0.039	−0.016	0.042	0.293	0.007	−0.005	−0.002
Household size	0.169∗∗∗	0.045	−0.030∗∗∗	−0.001	0.022∗∗∗	0.009∗∗∗	−0.146∗∗∗	0.056	−0.025∗∗∗	0.019∗∗∗	0.006∗∗
Education	−0.017	0.026	0.003	0.000	−0.002	−0.001	0.050	0.033	0.008	−0.006	−0.002
Off-farm income	−0.888∗∗∗	0.166	0.157∗∗∗	0.004	−0.115∗∗∗	−0.046∗∗∗	0.780∗∗∗	0.217	0.132∗∗∗	−0.099∗∗∗	−0.033∗∗∗
Training	−0.045	0.258	0.008	0.000	−0.006	−0.002	0.792∗∗	0.343	0.134∗∗	−0.101∗∗	−0.033∗∗
Irrigation	−0.428∗	0.237	0.076∗	0.002	−0.055∗	−0.022∗	0.912∗∗∗	0.314	0.154∗∗∗	−0.116∗∗∗	−0.038∗∗
Livestock ownership	−0.207	0.288	0.037	0.001	−0.027	−0.011	0.635∗	0.355	0.108∗	−0.081∗	−0.027∗
Self-confidence	−0.188∗	0.112	0.033∗	0.001	−0.024∗	−0.010	0.263∗	0.150	0.045∗	−0.034∗	0.011
Hope	−0.003	0.109	0.001	0.000	0.000	0.000	0.345∗∗	0.141	0.058∗	−0.044∗	−0.014∗
Optimism	0.055	0.109	−0.010	0.000	0.007	0.003	0.106	0.134	0.018	−0.013	−0.004
Resilience	−0.364∗∗∗	0.110	0.064∗∗∗	0.002	−0.047∗∗∗	−0.019∗∗∗	0.099	0.136	0.017	−0.013	−0.004

/cut1	−11.194	1.685					5.293	2.104			
/cut2	−9.316	1.647					7.579	2.118			
/cut3	−6.920	1.627									

Pseudo R^2^	0.11						0.11				
Log Likelihood	−340.081						−192.447				
Likelihood Ratio test	Chi^2^(14) = 83.43, p-value = 0.000						Chi^2^(14) = 49.83, p-value = 0.000				
Hosmer-Lemeshow test	Chi^2^ = 18.460, p-value = 0.732						Chi^2^ = 13.818, p-value = 0.539				
Lipsitz test	Chi^2^ = 11.026, p-value = 0.200						Chi^2^ = 11.548, p-value = 0.173				
Durbin-Wu-Hausman test	Chi^2^(1) = 0.000, p-value = 0.988						Chi^2^(1) = 0.132, p-value = 0.717				
Approximate Likelihood-Ratio test	Chi^2^(28) = 36.17, p-value = 0.138						Chi^2^(14) = 19.10, p-value = 0.161				
Multicollinearity test	Mean VIF = 1.20						Mean VIF = 1.20				

**Note**: ∗∗∗, ∗∗, and ∗ indicate the level of significance at 1 %, 5 %, and 10 %, respectively; FS, food secure; FI, food insecure.

The direction of the relationship between the explanatory variable and dependent variable was indicated by the sign of the estimated coefficient. For instance, a positive coefficient implied that an increase in a certain explanatory variable was associated with an increase in household food insecurity and nutrition security as measured by the HFIAP and FCS, respectively, *ceteris paribus*. A negative coefficient implied that an increase in a variable was associated with a decrease in household food insecurity and nutrition security. However, the estimated coefficient of the ordered logit model only provides the direction of the impact of the explanatory variable on the dependent variable and does not represent the actual magnitude of change or likelihood [70]. Hence, the marginal effects were also reported to show the expected change in the likelihood of being food secure, mildly, moderately, and severely food insecure, and of being in the poor, borderline, and acceptable food consumption group for a one-unit change in the explanatory variable.

The results showed that two explanatory variables capturing the role of fruit trees in food insecurity and nutrition security were statistically significant. The relationship between growing fruit trees and household food insecurity was negative (−1.077). The marginal effects' results showed that those who grow fruit trees had a 19.0% higher probability of being food secure than those who did not grow fruit trees. The probability of being moderately and severely food insecure was 14.0% and 5.5% lower, respectively, for those who grow fruit trees. The relationship between growing fruit trees and household nutrition security was positive (1.008). The probability of consuming acceptable diets increased by 17.1%, while the probability of being at the borderline and consuming poor diets decreased by 12.9% and 4.2%, respectively. This implies that households involved in fruit farming are more likely to have better access to food and nutrition. During the survey, some respondents indicated that they consume fruits produced on their farmland. For instance, they spread home-produced avocado on bread instead of store-bought spreads such as peanut butter. According to Omotayo and Aremu [13], nutritionally sufficient or healthy diets include fruits. Similarly, Bhebhe et al. [17] mentioned that tree resources contribute to households’ diverse diets. In the study locations, some households produced multiple types of fruits with different fruiting seasons. This improves food sustainability due to the availability of fruits in various seasons of the year. Moreover, fruit trees contribute to food insecurity reduction through income generation [16]. Some households indicated that they use the income generated from the sale of tree resources to buy food items.

The findings also showed a significant positive relationship between the consumption of wild fruits and food insecurity (0.892). The probability of being food secure decreased by 15.8% for households consuming wild fruits, while the probability of being moderately and severely food insecure increased by 11.6% and 4.6%, respectively. That is, a rise in the consumption of wild fruits was associated with an increase in household food insecurity. This is in line with Chakona and Shackleton [71], who found that households who consumed wild foods had a higher HFIAS score in their study. They argued that the consumption of wild foods tends to be higher among low-income households that cannot afford to purchase enough food. Ngidi [72] also reported that consumption of wild foods is a coping strategy for households with low dietary diversity, and who struggle to access food due to increasing food prices.

Three of the psychological capital variables (self-confidence, hope, and resilience) were statistically significant. The results showed that the association between self-confidence and household food insecurity and nutrition security was positive. The probability of being food secure and consuming acceptable diets increased by 3.3% and 4.5%, respectively. That is, confident individuals were less likely to experience food and nutrition insecurity. This is consistent with Jomaa et al. [73], who found that caregivers with high self-confidence had lower odds of household food insecurity. They reported that caregivers in food-insecure households were less confident in their abilities to choose the best-priced vegetables and fruits, purchase and cook healthy foods for their families on a budget, and stick to their grocery list compared to those in food-secure households. This suggests that increased confidence in managing food resources among rural households is associated with a lower risk of food and nutrition insecurity. Armstrong et al. [74] also demonstrated the essential role that confidence plays in improving household food and nutrition security.

The marginal effects’ results showed that the probability of consuming poor diets and being at the borderline for hopeful individuals decreased by 1.4% and 4.4%, respectively. This is in line with [75], who indicated that hopeful individuals can identify different ways to reduce nutrition insecurity even when facing difficult life events. These results suggest that a future-oriented mindset may enable rural households to prioritize their nutritional needs, explore alternative food sources, and adopt better budgeting strategies, even amidst financial or environmental constraints. Moreover, they highlight the psychological dimension of nutrition security, emphasizing the need for policies that promote mental well-being alongside interventions aimed at building sustainable and resilient food systems.

The relationship between resilience and household food insecurity status was negative and statistically significant. The probability of being moderately and severely food insecure decreased by 4.7% and 1.9%, respectively. These findings are in line with Egamberdiev et al. [27], who reported that resilient households are less likely to suffer from food insecurity. In the context of food insecurity, resilience refers to the household's capacity to bounce back from shocks and stresses such as crop failure, high food and agricultural inputs prices, job loss, death of the family breadwinner, livestock theft, floods, drought, and storm [[76], [77], [78]]. According to Chipfupa et al. [37], resilience is associated with the ability or possession of agricultural and non-agricultural assets. Several studies [76,79] have found a positive relationship between access to assets and rural household's resilience to food insecurity. This implies that rural households also require physical assets such as tractors, water tanks, and watering cans, among others, to be resilient to food insecurity.

The other three independent variables also had a significant relationship with both food insecurity and nutrition security (i.e., household size, off-farm income, and access to irrigation). The marginal effects’ results showed that an additional member of the household decreased the probability of being food secure and consuming acceptable diets by 3.0% and 2.5%, respectively. That is, an increase in the household size results in the likelihood of being in the higher categories of food and nutrition insecurity, *ceteris paribus*. This is in line with previous studies that reported that a larger family size leads to food and nutrition insecurity [8,80,81]. Cele and Mudhara [82] indicated that larger families usually experience higher food demand which, in turn, increases the risk of food insecurity. Another possible reason is that most rural households have many young and unemployed family members, thus, struggle to achieve food and nutrition security due to financial constraints [8,17].

The results showed that households generating higher income from off-farm activities are less likely to be food and nutrition insecure. These findings are consistent with Maziya et al. [8]. An increase in off-farm income decreased the likelihood of being moderately and severely food insecure by 11.5% and 4.6%, respectively. Moreover, the likelihood of consuming poor diets and being on the borderline decreased by 3.3% and 9.9%, respectively. The high-income households are more likely to purchase preferred, diverse, and nutritious food items and consume acceptable diets [80]. According to Anang et al. [83], access to off-farm income enables farming rural households to stabilize household income and reduce food insecurity associated with declining agricultural production due to climate change. Bhebhe et al. [17] also reported that higher income improves food access and availability.

The probability of being moderately and severely food insecure for households with access to irrigation decreased by 5.5% and 2.2%, respectively, while the probability of consuming poor diets decreased by 3.8%. This implies that households with access to water for irrigation purposes were more likely to be in the lower categories of food and nutrition insecurity than those who had no irrigation access, *ceteris paribus*. These results are consistent with several studies that indicated the importance of access to water in reducing food insecurity, especially in rural communities where most households depend on farming for food consumption and income generation [41,80,84]. A study by Nounkeu and Dharod [85] revealed that limited water access raises the risk of food insecurity because it negatively impacts food availability, access, and utilization. Other studies also mentioned that access to reliable sources of water enhances the year-round productivity of livelihood strategies such as growing crops, raising livestock, and planting trees, and reduces the undernourishment levels [86,87].

An increase in access to agricultural training was associated with increasing the probability of consuming acceptable diets by 13.4%. These results align with the agency pillar, which emphasizes the importance of empowering individuals to make informed choices and actively shape their food security outcomes [23]. According to Bahta and Musara [55], access to training programs equips individuals with agricultural and financial management skills, which can positively impact food-related decision-making within the households and reduce food and nutrition insecurity. The findings also showed that a unit rise in the number of livestock owned reduced the likelihood of consuming poor diets by 2.7%. This is in line with Cele and Mudhara [82], who reported that livestock ownership contributes to food and nutrition insecurity reduction through the direct provision of eggs, milk, and meat. It also contributes indirectly through income provision. During the survey, some respondents indicated that they kept livestock such as goats and domestic chickens for consumption purposes.

## 5 Conclusions and recommendations

This study investigated the role of fruit trees in food insecurity and nutrition security of rural households. It was conducted in the KwaZulu-Natal province, South Africa. The results showed that two explanatory variables capturing the role of fruit trees on food insecurity and nutrition security (i.e., growing fruit trees and consumption of wild fruits) were statistically significant. Growing fruit trees reduced household food and nutrition insecurity. This suggests that households practicing fruit farming are more likely to have better access to food and consume acceptable diets. To improve the planting of fruit trees in rural households, this study recommends the dissemination of information on the benefits of fruit trees. The consumption level of wild fruits among the sampled rural households was low. This indicates a need for awareness campaigns promoting the utilization and consumption of locally available and indigenous wild fruits. Encouraging rural households to consume wild fruits may reduce food insecurity through improved dietary diversity. It may also reduce reliance on purchased food items. Moreover, encouraging them to sell wild fruits may improve their household income.

The findings also showed that access to irrigation and off-farm income reduced food and nutrition insecurity. Therefore, this study recommends the establishment, rehabilitation, and revitalization of irrigation projects in rural communities. Given the rising water scarcity, training programs on using irrigation water productively and managing small-scale irrigation schemes are also required. This should be supported by value chain development and improved market access. Moreover, creating opportunities for off-farm income-generating activities is recommended. This could include training programs focused on enterprise development and increased investment in entrepreneurship, particularly for rural youth. Confident, hopeful, and resilient individuals were less likely to experience household food and nutrition insecurity. These results indicate that psychological capital plays a vital role in food and nutrition insecurity reduction. Nutrition-related training programs and workshops are recommended to enhance self-confidence in managing food resources. These programs can cover topics such as shopping strategies, budgeting, food selection, food preparation, and consumption of wild foods. This may also improve the consumption of acceptable food groups and the quality of food utilization in resource-poor communities.

Strategies related to improving rural households’ access to physical assets such as tractors, water tanks, and watering cans are suggested to enhance resilience to food insecurity. This study also recommends the collaboration of government, research and academia, private sector, civil society organizations and non-state actors, policymakers, politicians, and farming rural households to transform food systems and reduce food and nutrition insecurity. The involvement of local leaders, such as ward councillors and chiefs, is important because they play a vital role in the mobilization of resources at a local level. Moreover, the involvement of farming rural households may improve their ability to exercise their voices and participate in shaping local food systems. That is, it may contribute to the attainment of food agency. Future studies need to apply the theory of psychological capital in its entirety to household food consumption research. Moreover, these studies should use a data collection method that closely aligns with a revealed preference approach to measure the four constructs of psychological capital.

## 6 Limitations

In this study, the PCA-derived psychological capital indices had a Cronbach’s alpha value of 0.47 (see Appendix A), which is below the generally accepted threshold of 0.70. The low internal consistency or reliability may be partly due to the limited number of items, as only eight questions were used to measure psychological capital. To improve internal consistency, future studies should consider increasing the number of questions or items measuring each psychological capital construct. These studies should first pre-test the questions and conduct a pilot analysis to ensure the reliability and validity of the indices before proceeding with the main data collection and analysis.

## CRediT authorship contribution statement

**Fortunate Nosisa Zaca:** Writing – original draft, Visualization, Methodology, Investigation, Formal analysis, Data curation, Conceptualization. **Unity Chipfupa:** Writing – review & editing, Supervision. **Temitope Oluwaseun Ojo:** Writing – review & editing. **Lavhelesani Rodney Managa:** Writing – review & editing, Visualization. **Tafadzwanashe Mabhaudhi:** Writing – review & editing. **Rob Slotow:** Writing – review & editing. **Mjabuliseni Simon Cloapas Ngidi:** Writing – review & editing, Supervision.

## Data availability statement

The data presented in this study are available on request from the corresponding authors. The data are not publicly available due to confidentiality.

## Funding

This research was partially funded by the Wellcome Trust's Climate and Health Programme as part of the Sustainable and Healthy Food Systems - Southern Africa (SHEFS-SA) Project [grant number: 227749/Z/23/Z]. For the purpose of open access, the authors have applied for a CC BY public copyright license to any author-accepted manuscript version arising from this submission. The National Research Foundation of South Africa [grant number: PMDS22062125472] and the University of KwaZulu-Natal's WoodRIGHTS Flagship Project also funded this research.

## Declaration of competing interest

The authors declare that they have no known competing financial interests or personal relationships that could have appeared to influence the work reported in this paper.

## Figures and Tables

**Table A1:** Principal component analysis results for psychological capital measures.

Variables	Principal components			
	PC_1_ – Self-confidence	PC_2_ – Hope	PC_3_ – Optimism	PC_4_ – Resilience
**Hope**				
Given the current farming constraints, do you believe that there is a potential to turn things around?	0.303	0.481	−0.093	0.476
Given the current unemployment rate, do you believe that youth have the potential to start businesses and create more jobs?	−0.009	**0.742**	−0.169	−0.040
**Resilience**				
If your crops are affected by pests, will you seek to raise money to buy effective pesticides or pest-resistant crops in the next season?	0.024	−0.101	0.069	**0.927**
If your business has been making a loss for the past three years, will you continue with it and consult a business advisor or successful business owner?	0.186	**0.652**	0.222	−0.024
**Self-confidence**				
Do you believe that you are most likely to be nominated by others as a leader in the community?	**0.875**	0.130	0.002	0.081
Would you accept the nomination if you were nominated as a committee member in an organization?	**0.879**	0.057	0.001	0.031
**Optimism**				
When faced with poor yields and struggling to meet basic needs, would you continue farming and see the constraints as temporary?	−0.080	0.318	**0.736**	0.178
If the government introduces a new land consolidation program, are you most likely going to refuse the compensation and keep your land?	0.068	−0.323	**0.731**	−0.113

*Eigenvalue*	2.06	1.21	1.16	1.00
*% of variance*	25.71	15.15	14.47	12.51
*Cumulative % of variance*	25.71	40.86	55.33	67.84

**Note:** Only component loadings greater than |0.50| were used to name the principal components; KMO = 0.61; Bartlett's Test of Sphericity Chi^2^ = 257.17, p-value = 0.000; and Cronbach’s alpha = 0.47.
