# Supplementary material for: The role of fruit trees in reducing food insecurity and improving nutrition security of rural households: A case study of the KwaZulu-Natal province, South Africa
Source: J Agric Food Res. Author manuscript; Available in PMC 2026 Feb 24. (PMC12144314; doi:10.1016/j.jafr.2025.101883)
Supplement: Appendix A [file EMS212476-supplement-Appendix_A.pdf]

Appendix A

Table A1
Principal component analysis results for psychological capital measures.

Variables	Principal components			
	PC_1 – Self-confidence	PC_2 – Hope	PC_3 – Optimism	PC_4 – Resilience
**Hope**				
Given the current farming constraints, do you believe that there is a potential to turn things around?	0.303	0.481	−0.093	0.476
Given the current unemployment rate, do you believe that youth have the potential to start businesses and create more jobs?	−0.009	**0.742**	−0.169	−0.040
**Resilience**				
If your crops are affected by pests, will you seek to raise money to buy effective pesticides or pest-resistant crops in the next season?	0.024	−0.101	0.069	**0.927**
If your business has been making a loss for the past three years, will you continue with it and consult a business advisor or successful business owner?	0.186	**0.652**	0.222	−0.024
**Self-confidence**				
Do you believe that you are most likely to be nominated by others as a leader in the community?	**0.875**	0.130	0.002	0.081
Would you accept the nomination if you were nominated as a committee member in an organization?	**0.879**	0.057	0.001	0.031
**Optimism**				
When faced with poor yields and struggling to meet basic needs, would you continue farming and see the constraints as temporary?	−0.080	0.318	**0.736**	0.178
If the government introduces a new land consolidation program, are you most likely going to refuse the compensation and keep your land?	0.068	−0.323	**0.731**	−0.113
Eigenvalue	2.06	1.21	1.16	1.00
% of variance	25.71	15.15	14.47	12.51
Cumulative % of variance	25.71	40.86	55.33	67.84

Note: Only component loadings greater than |0.50| were used to name the principal components; KMO = 0.61; Bartlett's Test of Sphericity Chi^2 = 257.17, p-value = 0.000; and Cronbach's alpha = 0.47.
Source: Authors' own work
